# Excisions of Head of Os Humeri for Gun Shot Wounds

**Published:** 1871-08

**Authors:** Charles M. Clark

**Affiliations:** Chicago


ARTICLE III.—*Excisions of Head of Os Humeri for Gun Shot Wounds.* By CHARLES M. CLARK, M.D., Chicago.

(Continued from page 331.)

CASE III.

Nathan E. Davis, 2d Lieut., Co. F. 39th Ill. Vols., wounded October 13, 1864, by rifle ball, which entered at upper and outer third of right humerus, passing downwards and inwards, making its exit at the middle third of arm.

The bone was extensively comminuted, with a corresponding degree of mutilation to the soft tissues. He was placed upon the operating table, from the ambulance, immediately upon its arrival from the field, and chloroform administered.

The following operation was then performed; Longitudinal incision was commenced at the acromion and carried down on dorsum of arm, to the extent of seven inches, through to the bone, care being taken to leave as much of the periosteum as was valuable, when removing the fragments, which amounted to twenty-seven pieces in all. Provision was also made to isolate the circumflex nerve, which, in this particular case, was of enormous size.

After the removal of all the "debris" of bone, blood clots, etc., up to the capsule of the joint, it (the capsule) was divided and the head of bone removed, which was found fractured in its longitudinal diameter. Then section was made of the lower and remaining shaft at the point of perfect integrity, and after a thorough cleansing, the lips of the wound were brought together with the interrupted silk suture, and dressed with adhesive straps—compress of lint—splints and bandage, and the man carried to a bed.

The following morning he appeared cheerful and expressed himself as feeling very comfortable and greatly relieved, and could have slept some had it not been for the constant attention of the nurse in the application of cold water to the limb.

Reaction was fully established, and he called for his breakfast, which was eaten with relish.

The wound had bled some throughout the night, but had no depressing effect, and, in fact, was nothing more than is usual in this class of operations, from venous congestion and the oozing from so large a cavity as this was, there having been fully five inches of bone removed, including the "os humeri."

He was conveyed to general hospital at Fortress Monroe, by hospital transport, the following day.

In a few days the wound was attacked with hospital gangrene, (the great scourge in Chesapeake hospital at that time) and in a few days he died.

Previous to his wound, and at the time of being wounded, he was suffering with chronic diarrhœa, and had but lately returned from a furlough given him on that account. His system was vitiated, and the vital powers much below a normal standard.

### CASE IV.

Horace W. Stilwell, private, Co. B., 142d New York Vols., wounded, October 27, 1864, by conoidal bullet, which entered right shoulder anterior to head of bone, and passed obliquely downward and backward, lodging just underneath the skin at the middle third of the arm.

The bone was greatly shattered, and the capsule of the joint extensively lacerated, with free discharge of synovial fluid.

Chloroform was administered, and the head of bone, together with six inches of the shaft, removed, by an operation similar to the ones already described.

He rallied well from the operation, and on the following day was sent to general hospital at Fortress Monroe.

I have never learned the result of this case from any authentic source.

### CASE V.

William Mahon, private, Co. —, 67th Ohio Vol. Infantry, wounded, June 2, 1864, by rifle ball, which entered the left shoulder, on its dorsal surface, passing transversely through the joint—splitting the head of bone and comminutely fracturing the neck and shaft, finally lodging just underneath the integument of inner surface of arm—or rather within the triangle of the axilla. The man was not brought from the field until nine o'clock P. M., and it was fully midnight before he was placed upon the table. Chloroform was given, and a full and free examination made by many of the surgeons attached to the operating staff. The decision arrived at was, that the operation for excision of the "os humeri" should be performed.

This was the first operation for excision of the shoulder joint, and, in fact, the first excision of any character in the hospitals of the Army of the James, and it was proceeded with under the most adverse circumstances, as far as light was concerned, for our supply of candles had given out, with the exception of one which was called into use at this time.

Dr. H. P. Babcock, U. S. N., then on shore, from the U. S. gunboat "Agawam," and who during the day had rendered valuable service, kindly assisted me in the operation, together with my usual table assistants.

The operation consisted in the same steps as heretofore described, and occupied fully an hour's time, by reason of the unfavorable conditions of insufficient light and inexperience with the operation, it being the first operation of the kind that I had attempted.

Great care was exercised in preserving the periosteum and in the dressing of the arm after the operation.

The following day he was placed on the hospital transport, "Hero of Jersey," and conveyed to Chesapeake hospital.

Six months afterwards, I was told by his regimental surgeon (Dr. James Westfall), that he made a good recovery—had been discharged from the service and was living at Toledo, Ohio, with a useful arm.

